# Circ_0119872 promotes uveal melanoma development by regulating the miR-622/G3BP1 axis and downstream signalling pathways

**DOI:** 10.1186/s13046-021-01833-w

**Published:** 2021-02-12

**Authors:** Shuting Liu, Liang Chen, Hua Chen, Kangkang Xu, Xi Peng, Mingchang Zhang

**Affiliations:** 1 Department of Ophthalmology, Union Hospital, Tongji Medical College, Huazhong University of Science and Technology, Wuhan, 430022 HB China; 2 Department of Urology, Union Hospital, Tongji Medical College, Huazhong University of Science and Technology, Wuhan, 430022 HB China

**Keywords:** Circ_0119872, Uveal melanoma, G3BP1, Wnt/β-catenin, mTOR

## Abstract

**Background:**

The abnormal expression of circular RNAs (circRNAs) in uveal melanoma (UM) has been revealed, but the specific underlying molecular mechanism of their association with UM development has not been fully explored.

**Methods:**

The levels of circ_0119872, G3BP1 and miR-622 in UM cell lines and tissues were determined by quantitative real-time PCR (qRT-PCR) and western blotting assays. In vitro and in vivo assays were performed to investigate the function of circ_0119872 in the tumorigenesis of UM cells. The relationships among circ_0119872, miR-622 and G3BP1 were predicted using bioinformatic tools and verified by RNA-FISH, RNA pull-down and dual-luciferase reporter assays. The effects of circ_0119872 on Wnt/β-catenin and mTOR signalling pathways were determined by gene set enrichment analysis (GSEA) and western blotting.

**Results:**

We found that circ_0119872 is upregulated in UM cell lines and tissues. Moreover, overexpression of circ_0119872 promotes the malignancy of UM cells, while silencing of circ_0119872 inhibits it. In addition, circ_0119872 can directly interact with miR-622 as a miRNA sponge that regulates the expression of the miR-622 target gene G3BP1 as well as downstream Wnt/β-catenin and mTOR signalling pathways.

**Conclusions:**

Circ_0119872 may act as an oncogene in UM through a novel circ_0119872/miR-622/G3BP1 axis, activating the Wnt/β-catenin and mTOR signalling pathways, which in turn may provide potential biomarkers and therapeutic targets for the management of UM.

**Supplementary Information:**

The online version contains supplementary material available at 10.1186/s13046-021-01833-w.

## Background

Uveal melanoma (UM), which arises from melanocytes in the uvea and comprises the pigmented tissues of the iris, ciliary body and choroid, is the second most common type of melanoma [1]. Long-term mortality from UM slightly exceeds 50% [2], and because it appears to be both prone to early metastasis and resistant to available treatments once disseminated, it is unlikely that we can considerably reduce mortality in the near future. Therefore, to develop novel and effective therapeutic approaches, understanding the molecular mechanism of UM development and progression is of great importance.

Circular RNAs (circRNAs) are a subclass of non-coding RNAs (ncRNAs) that lack free 3′ and 5′ ends and thus exist as continuous loop RNAs. They are produced by a non-canonical splicing event called back-splicing. For a long time, circular transcripts were considered aberrant splicing by-products [3]. However, high-throughput transcriptome sequencing and detailed molecular characterization of individual circRNAs have revealed their ubiquitous expression.

Increasing evidence suggests that circRNAs are functional molecules. To date, circRNAs have been implicated in several human diseases, including diabetes mellitus, neurological disorders, cardiovascular diseases, chronic inflammatory diseases and cancer, and have been shown to accumulate during ageing [4, 5].

In the present study, we identified a novel circRNA, circ_0119872, that acts as an oncogene in UM. The expression of circ_0119872 is significantly upregulated in UM tissues and cell lines and is positively associated with UM progression by sponging miR-622 to influence the expression of G3BP1 and the activity of Wnt/β-catenin and mTOR signalling pathways. Circ_0119872 may exert regulatory functions and serve as a target for UM treatment.

## Methods

### Cell culture

The cell lines used in this study, including the human retinal pigment epithelial cell line (APRE-19) and UM cell lines (SP6.5, VUP, OCM-1, 92–1, OCM-1A, MUM-2B, and OM431), were obtained from the Cell Bank of the Chinese Academy of Sciences (Shanghai, China). All the cell lines were maintained in DMEM (Gibco, CA, USA) supplemented with 10% foetal bovine serum (FBS) and 1% penicillin/streptomycin (Gibco, CA, USA), and incubated with 5% CO_2_ at 37 °C.

### Tissue specimens

UM tissues and adjacent normal tissues were obtained from the Department of Ophthalmology, Huazhong University of Science and Technology Affiliated Union Hospital and stored in liquid nitrogen. All experiments involving human tissues were carried out in accordance with the Declaration of Helsinki and approved by the institutional research ethics committee.

### Quantitative real-time PCR (qRT-PCR)

Total RNA of cell lines or fresh tissues was extracted using TRIzol reagent (Invitrogen, CA, USA) according to the manufacturer’s protocol and was reverse transcribed using the PrimeScript RT Reagent Kit (Takara, Japan). Subsequently, qRT-PCR was performed on the StepOnePlus Real-Time PCR System (Life Technologies, Carlsbad, CA). GAPDH and U6 were used as internal controls, and the 2^–ΔΔCT^ method was used to evaluate the relative expression of circRNA, miRNA, and mRNA. The sequences of primers used in this study are listed in Additional file 1: Table S1.

### Western blotting

Cells and tissue samples were lysed in RIPA lysis buffer with 1% PMSF. The BCA Protein Assay Kit (Thermo Scientific, MA, USA) was utilized to measure the protein concentration of each sample. Then, the proteins were separated by electrophoresis in SDS-PAGE gels and transferred to PVDF membranes. After being blocked in 5% milk for 1 h at room temperature, PVDF membranes were incubated with the primary antibodies overnight at 4 °C and the corresponding species-specific secondary antibodies for 1 h at room temperature. Finally, the protein bands were detected by chemiluminescence using an electrochemiluminescence (ECL) system. The following antibodies were used for western blotting analysis: anti-Alpha Tubulin (Cat. No. 66031–1-Ig, Proteintech, USA), anti-G3BP1 (Cat. No. 66486–1-Ig, Proteintech, USA), anti-Beta Catenin (Cat. No. 51067–2-AP, Proteintech, USA), anti-Cyclin D1 (Cat. No. 60186–1-Ig, Proteintech, USA), anti-C-MYC (Cat. No. 10828–1-AP, Proteintech, USA), anti-Beta Actin (Cat. No. 66009–1-Ig, Proteintech, USA), anti-P84 (Cat. No. 10920–1-AP, Proteintech, USA), anti-mTOR (Cat. No. 66888–1-Ig, Proteintech, USA), anti-p-mTOR (Cat. No. 67778–1-Ig, Proteintech, USA), anti-4E-BP1 (Cat. No. ab131453, Abcam, UK), anti-p-4E-BP1 (Cat. No. ab27792, Abcam, UK), anti-S6K1 (Cat. No. ab9366, Abcam, UK) anti-p-S6K1 (Cat. No. ab59208, Abcam, UK), HRP-conjugated secondary goat anti-rabbit antibody (Cat. No. SA00001–2, Proteintech, USA), and HRP-conjugated secondary goat anti-mouse antibody (Cat. No. SA00001–1, Proteintech, USA).

### Plasmids and transfection

Human circ_0119872 or G3BP1 cDNA was amplified by PCR and cloned into the pcDNA3.1(+) vector to construct the overexpression plasmid. ShRNAs targeting G3BP1 were designed and cloned into GV248 (GeneChem, Shanghai, China), while shRNAs targeting circ_0119872 were cloned into GV102 (GeneChem, Shanghai, China). Plasmids were transfected with Lipofectamine 2000 reagent (Invitrogen, CA, USA) according to the manufacturer’s instructions, and cell lines transfected with GV248 were selected with 2 μg/ml of puromycin for 30 days, while cell lines transfected with pcDNA3.1(+) or GV102 were selected with 2.5 μg/ml of G418 for 30 days.

The miRNA mimics and inhibitors were purchased from RiboBio (Guangzhou, China) and transfected into cells with RNAiMAX transfection reagent (Invitrogen, CA, USA).

### RNase R treatment

For RNase R treatment, 2 μg of total RNA extracted from UM cells was incubated with or without 3 U/μg RNase R (Epicenter, WI, USA) at 37 °C for 15 min. Subsequently, the RNA treated with RNase R was purified using the RNeasy Mini Kit (QIAGEN, Dusseldorf, Germany) according to the manufacturer’s protocol. And then, purified RNA from each group was reverse transcribed to cDNA using the PrimeScript RT Reagent Kit (Takara, Japan). On the one hand, the levels of circ_0119872 and RASGRP3 were detected by qRT-PCR on the StepOnePlus Real-Time PCR System (Life Technologies, Carlsbad, CA). On the other, the cDNA of circ_0119872 and RASGRP3 was amplified by PCR, and the products were observed using 1.5% agarose gel electrophoresis.

### RNA fluorescence in situ hybridization (RNA-FISH)

Probes for circ_0119872 and miR-622 used in the RNA-FISH assay were synthesized by RiboBio (Guangzhou, China). In short, UM cells were fixed with 4% paraformaldehyde and permeabilized with 0.5% Triton X-100. Then, the cells were hybridized with a specific probe at 37 °C overnight. After washing with saline sodium citrate (SSC), the cells were counterstained with DAPI for 30 min at room temperature. All fluorescence images were captured using a Nikon A1Si laser scanning confocal microscope (Nikon Instruments Inc., Japan).

### Pull-down assay with biotinylated circRNA probe

UM cells (1 × 10^7^) were fixed with 1% paraformaldehyde and then lysed in lysis buffer. After that, 20 μl of supernatant was reserved as input, and the remaining supernatant was mixed with biotin-labelled probes at room temperature for 4 h. Subsequently, all samples were incubated with M-280 streptavidin magnetic beads (Invitrogen, CA, USA) at 4 °C overnight. The following day, beads were washed thoroughly with wash buffer, and proteinase K was used to reverse the formaldehyde cross-linking. The RNA complexes bound to the beads were isolated with the RNeasy Mini Kit (QIAGEN, Dusseldorf, Germany) and were analysed by qRT-PCR.

### Pull-down assay with biotinylated miRNA probe

UM cells were transfected with biotin-labelled miRNA mimics or nonsense control. After 48 h of transfection, cells were harvested and sonicated. Fifty microlitres of each lysate was used as input, and the remaining lysate was incubated with M-280 streptavidin magnetic beads (Invitrogen, CA, USA) at 4 °C on a rotator overnight. The next day, the beads were washed with wash buffer. The RNAs bound to magnetic beads were purified with the RNeasy Mini Kit (QIAGEN, Dusseldorf, Germany) and analysed by qRT-PCR.

### Dual-luciferase reporter assay

For dual-luciferase reporter assay, UM cells were seeded in 24-well plate 24 h before transfection. Then, the cells were co-transfected with psiCHECK-2 G3BP1–3’UTR wide type (G3BP1–3’UTR) or mutant (G3BP1–3’UTR-Mut #1, #2, and #3) reporter vector and miR-622 mimics (20 nM) to examine the miRNA binding ability. After transfection for 48 h, the firefly and renilla luciferase activities were measured with Dual-Luciferase Reporter Assay System (Promega, WI, USA) according to the manufacturer’s protocol.

### In vivo growth and metastasis assays

Studies involving animals were approved by the Ethics Committee of Tongji Medical College of Huazhong University of Science and Technology. BALB/c-nu mice (3–4 weeks of age) were purchased from the Center of Experimental Animals of Tongji Medical College of Huazhong University of Science and Technology.

For the tumour formation assay, mice were randomly divided into groups (*n* = 5/group), and 2 × 10^6^ cells were injected subcutaneously into one side of each mouse. Every 3 days, tumour volumes were measured using an external calliper and calculated by the eq. (L × W^2^)/2. On day 30, animals were euthanized, and tumours were excised and weighed. The digital single-lens reflex camera (D610, Nikon Corporation, Japan) was used to acquire images of xenografts in nude mice.

For the metastasis assay, 2 × 10^6^ cells were injected into the tail-vein of each mouse. On day 50, all animals were euthanized by cervical dislocation, and lungs were excised and imaged using a In Vivo Optical Imaging System (In Vivo FX PRO, Bruker Corporation, USA).

### Human umbilical vein endothelial cell (HUVEC) tube formation assay

To perform the tube formation assay, precooled Matrigel (Becton, Dickinson and Company, NJ, USA) was coated into each well of a 24-well plate and polymerized for half an hour at 37 °C. HUVECs (1 × 10^5^) and 500 μl medium from different groups of UM cells were added to each well and incubated at 37 °C under 5% CO_2_. Wells were observed every 2 h. At the proper time, capillary tube structures were imaged under a bright-field microscope.

### Cell counting kit-8 (CCK-8) assay

Cell proliferation was quantified with the CCK-8 assay according to the manufacturer’s protocol. Briefly, cells (3 × 10^3^/well) were seeded into a 96-well plate and cultured at 37 °C. After incubation with 10 μl of CCK-8 reagent (DOJINDO, Kumamoto, Japan) for 1.5 h, the optical density was measured at 450 nm using a microtiter plate reader.

### 5-Ethynyl-2′-deoxyuridine (EdU) labelling

Cells were incubated with EdU reagent (RiboBio, Guangzhou, China.) for 2 h at 37 °C and then treated with ApolloR reaction cocktail according to the manufacturer’s instructions. Images were collected using fluorescence microscopy (Olympus, Japan).

### Migration and invasion assays

Cell migration and invasion were evaluated using Transwell chambers (Corning Life Sciences, MA, USA). For migration assay, 4 × 10^4^ cells suspended in 200 μl of serum-free medium were seeded in the upper chamber, and medium supplemented with 10% FBS was added to the lower chamber. For invasion assay, 8 × 10^4^ cells were seeded in Matrigel-precoated Transwell chambers. After incubation for 24 h, cells remaining on the top surface were removed, while cells migrated or invaded to the lower surface of the membrane were fixed and stained with 0.1% crystal violet, and counted under a light microscope.

### Statistical analysis

Statistical analysis was conducted with SPSS 16.0 software, and significance was analysed with Student’s t-test. Overall survival and recurrence-free survival curves were calculated by the Kaplan–Meier method and compared using the log-rank test. The cut-off point was defined as the median. In this study, *P* < 0.05 was considered statistically significant. Data from at least 3 independent experiments are expressed as the mean ± SD.

## Results

### Circ_0119872 is significantly upregulated in UM tissues

**Fig. 1:**
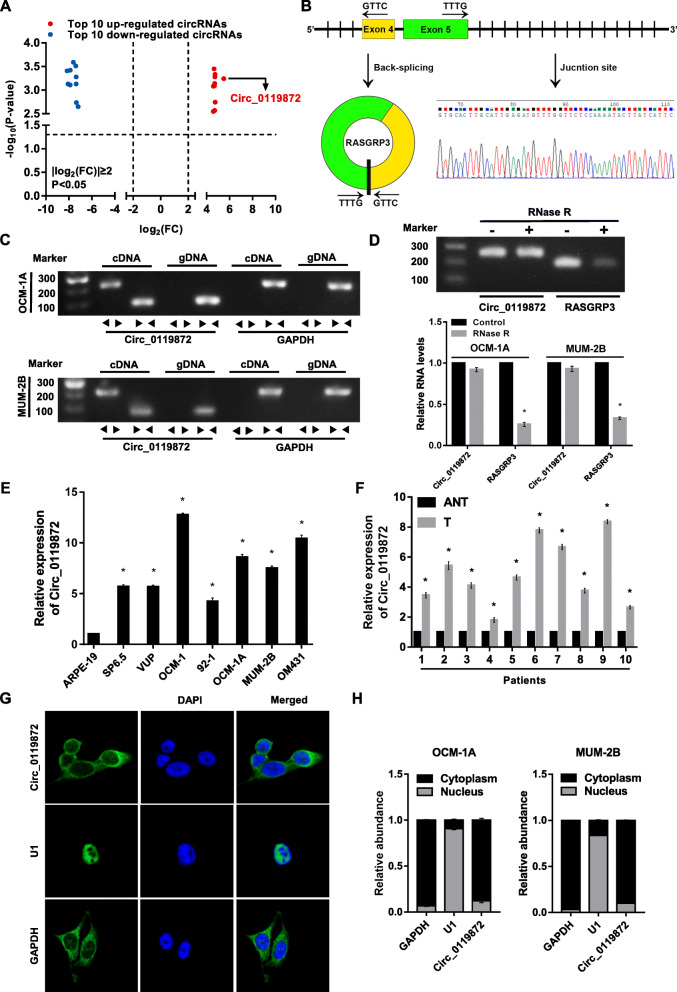
Identification of circ_0119872 in uveal melanoma (UM). **a** Top 10 significantly down-regulated and up-regulated circRNAs in UM tissues ranked by FC. **b** Schematic diagrams show that circ_0119872 is composed of exons 4 and 5 of RASGRP3. The existence of circ_0119872 was demonstrated by PCR, and its back-splicing junction was verified by Sanger sequencing. **c** PCR assay with divergent and convergent primers indicating that circ_0119872 is present in the OCM-1A and MUM-2B cell lines. GAPDH was used as a negative control. **d** The expression of and RASGRP3 after RNase R treatment in OCM-1A and MUM-2B cells. **e** The expression level of circ_0119872 in a human retinal pigment epithelial cell line (APRE-19) and UM cell lines (SP6.5, VUP, OCM-1, 92–1, OCM-1A, MUM-2B, OM431). **f** The expression level of circ_0119872 in 10 pairs of UM and adjacent non-tumour tissues. **g** and **h** RNA-FISH and RNA nucleus/cytoplasm separation assay indicates the location of circ_0119872. Nuclei are stained blue with DAPI. Circ_0119872 is stained green with FAM. Bar graphs show the statistical analysis of three independent experiments (* *P* < 0.05)

To identify the role of circRNAs in the development of uveal melanoma, differentially expressed circRNAs were acquired from the circRNA microarray data of a published study. In that research, a microarray was used to compare the expression profiles of circRNAs in five UM samples and five normal uvea tissues. In total, 50,579 circRNAs [|log_2_(FC)| ≥ 2.0; *P* < 0.05), of which 20,654 were upregulated and 29,925 were downregulated, were identified as differentially expressed between UM tissues and normal uvea tissues [6]. The top 10 significantly down-regulated and up-regulated circRNAs in UM tissues were summarized (Additional file 2: Table S2) and shown (Fig. 1a). Among them, circ_0119872 was upregulated most significantly in UM compared to normal tissues (Fig. 1a), so we chose the circ_0119872 for the following study. The genomic structure indicates that circ_0119872 is composed of exons 4 and 5 of the RASGRP3 gene. Subsequently, this circular product was amplified and confirmed by Sanger sequencing (Fig. 1b). We next designed convergent primers and divergent primers to amplify linear and circular RNA based on cDNA and genomic DNA (gDNA) from UM cells. Circ_0119872 was amplified only by the divergent primers from cDNA, and no amplification product was observed with gDNA (Fig. 1c). The results of the RNase R treatment assay indicate that circ_0119872 is much more resistant to RNase R than linear RASGRP3 mRNA (Fig. 1d).

To validate the expression pattern, we detected the level of circ_0119872 in UM cell lines, clinical UM tissues, and the corresponding normal control using qRT-PCR. A significant upregulation of circ_0119872 expression was observed in UM cell lines (Fig. 1e) and tumour tissues (Fig. 1f). Furthermore, RNA-FISH and RNA nucleus/cytoplasm separation assays were conducted to determine the cell distribution of circ_0119872, and the results showed that circ_0119872 was mainly localized in the cytoplasm (Fig. 1g and h).

### Circ_0119872 promotes cell proliferation and angiogenesis in vitro and tumour growth in vivo

**Fig. 2:**
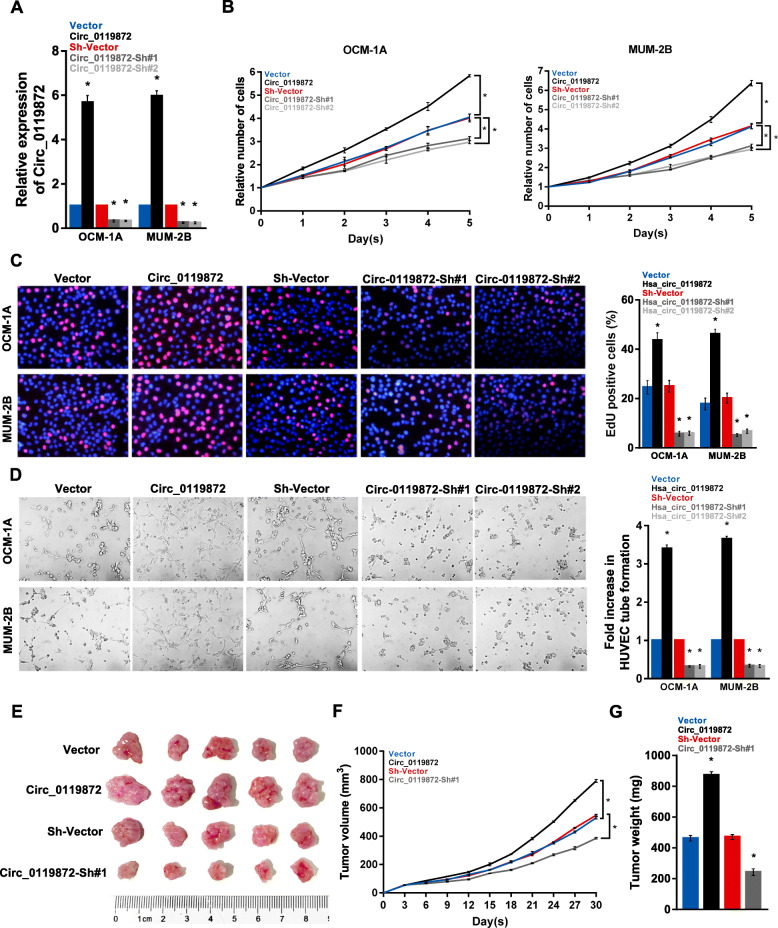
Circ_0119872 promotes the malignancy of UM cells in vitro and in vivo. **a** The expression levels of circ_0119872 in OCM-1A and MUM-2B cells stably transfected with circ_0119872 overexpression or knockdown vector and the corresponding negative controls were detected by qRT-PCR. **b** Circ_0119872 promotes proliferation in UM cells, as determined by the CCK-8 assay. **c** Representative micrographs (left panel) and quantification (right panel) of EdU incorporation in UM cells as indicated. DAPI was used as a DNA/nuclear stain. **d** Representative images (left panel) and quantification (right panel) of HUVECs cultured on Matrigel-coated plates with conditioned medium from UM cells. **e** Representative images of tumours from the xenograft model in nude mice. **f** Tumour volumes were measured every third day. **g** Tumour weight were measured on the day 30. Bar graphs show the statistical analysis of three independent experiments (* *P* < 0.05)

To investigate the biological role of circ_0119872 in UM, we performed gain-of-function assays by transfecting circ_0119872 overexpression (circ_0119872) or interference (circ_0119872-sh#1 and circ_0119872-sh#2) vectors into OCM-1A and MUM-2B cells (Fig. 2a). Ectopic expression of circ_0119872 promoted cell proliferation, while silencing of circ_0119872 expression inhibited the proliferation of both OCM-1A and MUM-2B cells (Fig. 2b and c). The results of the HUVEC tube formation assay also indicated that circ_0119872-transduced cells exhibited a significantly increased angiogenesis, while suppressing circ_0119872 had the opposite effect (Fig. 2d). However, circ_0119872 had no effect on cell migration or invasion according to the results of migration and invasion assays (Additional file 3: Fig. S1A).

To explore the impact of circ_0119872 on UM growth in vivo, we subcutaneously implanted circ_0119872-overexpressing or circ_0119872-knockdown UM cells into the flanks of athymic nude mice. The in vivo study showed that tumours in the circ_0119872-overexpressing group were significantly larger and heavier than those in the control group (Fig. 2e-g). In accordance with the migration and invasion assays, the result of in vivo metastasis assay also indicated that suppressing circ_0119872 could not affect the metastasis of UM cells (Additional file 3: Fig. S1B).

Overall, both in vitro and in vivo experiments suggest that circ_0119872 promotes UM cell proliferation and angiogenesis.

### Circ_0119872 directly interacts with miR-622 in UM cells

**Fig. 3:**
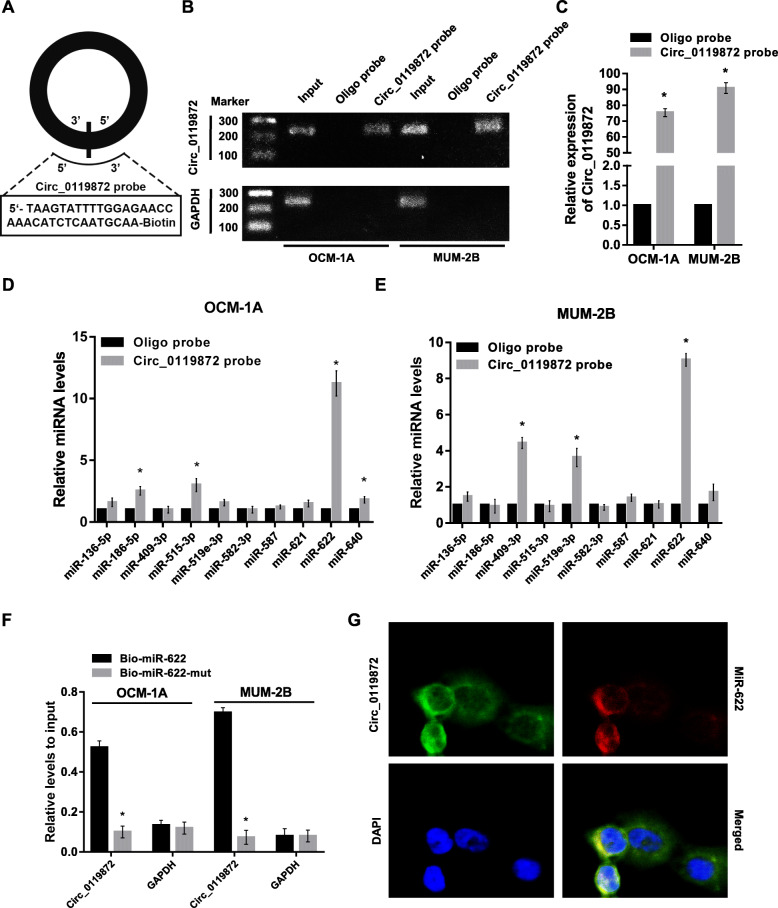
Circ_0119872 interacts with miR-622 in UM cells. **a** Schematic diagram displaying the probe specifically designed for circ_0119872. **b** and **c** Gel electrophoresis and qRT-PCR results showed that the circ_0119872 probe could specifically pull down the circular form of circ_0119872. **d** and **e** The relative expression levels of ten miRNA candidates pulled down by the circ_0119872 probe were detected by qRT-PCR. **f** Circ_0119872 was enriched by biotinylated wild-type miR-622 or its mutant, and qRT-PCR was used to determine the relative circ_0119872 levels. **g** RNA-FISH assay showed the colocalization between circ_0119872 and miR-622 in UM cells. MiR-622 probes were labelled with Cy3. Circ_0119872 probes were labelled with FAM. Nuclei were stained with DAPI. Data are represented as the mean ± SD of three independent experiments (* *P* < 0.05)

Previous studies have reported that circRNAs can influence the biofunctions of cancer cells by sponging miRNAs. To elucidate whether circ_0119872 functions through this mechanism in UM cells, we designed a specific biotin-labelled probe for circ_0119872 (Fig. 3a) and then identified ten candidate miRNAs that might be bound by circ_0119872 with specific target sites using the bioinformatic tool CircInteractome (https://circinteractome.nia.nih.gov/) [7]. As shown in Fig. 3b and c, circ_0119872 was specifically enriched by the circ_0119872 probe, which verified the efficiency and specificity of the pull-down assay. Then, the relative levels of the ten candidate miRNAs pulled down by circ_0119872 were evaluated. We found that only miR-622 was abundantly pulled down in both OCM-1A and MUM-2B cell lines (Fig. 3d and e). In turn, the enrichment of circ_0119872 in the miR-622 mutant-captured fraction (Bio-miR-622-mut) was largely diminished compared with that in the wild-type-captured fraction (Bio-miR-622) (Fig. 3f). Furthermore, RNA-FISH detection indicated that circ_0119872 and miR-622 were colocalized in the cytoplasm (Fig. 3g). Collectively, these results demonstrate that circ_0119872 can directly interact with miR-622 in UM cells and suggest that circ_0119872 functions as a miRNA sponge for miR-622.

### MiR-622 can reverse the effects of circ_0119872 on biofunction in UM cells

**Fig. 4:**
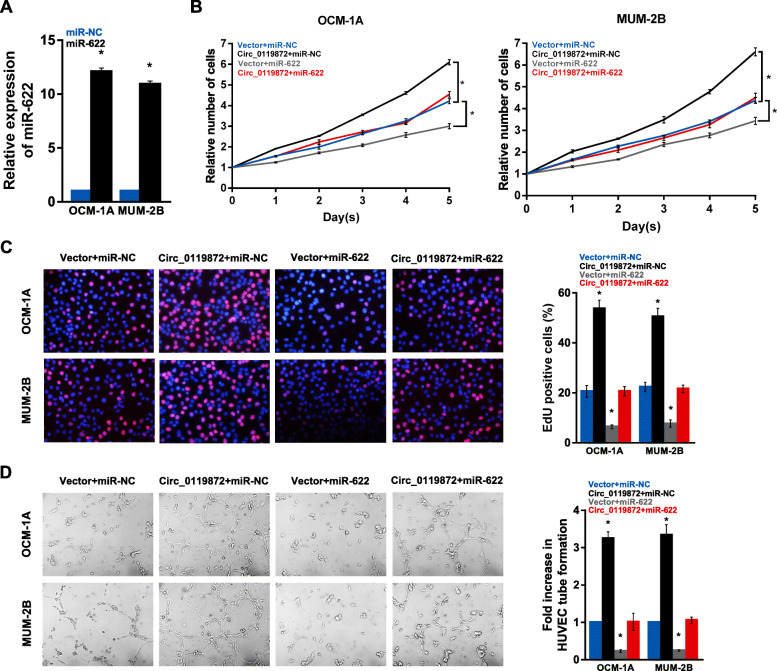
MiR-622 inhibits the malignancy of UM cells and reverses the promoting effects of circ_0119872. **a** The levels of miR-622 in OCM-1A and MUM-2B cells transfected with miR-622 mimics and the corresponding negative controls were detected by qRT-PCR. **b** and **c** CCK-8 and EdU assays demonstrate that circ_0119872 increases the proliferation of OCM-1A and MUM-2B cells, and when co-transfected with miR-622, the promoting effect is reversed. **d** HUVEC tube formation assay demonstrates that circ_0119872 increases the angiogenesis of OCM-1A and MUM-2B cells, and when co-transfected with miR-622, the promoting effect is reversed. Data are represented as the mean ± SD of three independent experiments (* *P* < 0.05)

To further confirm the co-regulatory effect of circ_0119872 and miR-622 on cell proliferation and angiogenesis, we upregulated circ_0119872 and then overexpressed miR-622 in UM cells (Fig. 4a). The results showed that miR-622 overexpression effectively abolished the promotive effect of circ_0119872 on cell proliferation and angiogenesis (Fig. 4b-d). These results indicate that miR-622 can act as a target of circ_0119872 to reverse the effects of circ_0119872 on biofunction in UM cells.

### MiR-622 directly targets G3BP1, which is upregulated in UM

**Fig. 5:**
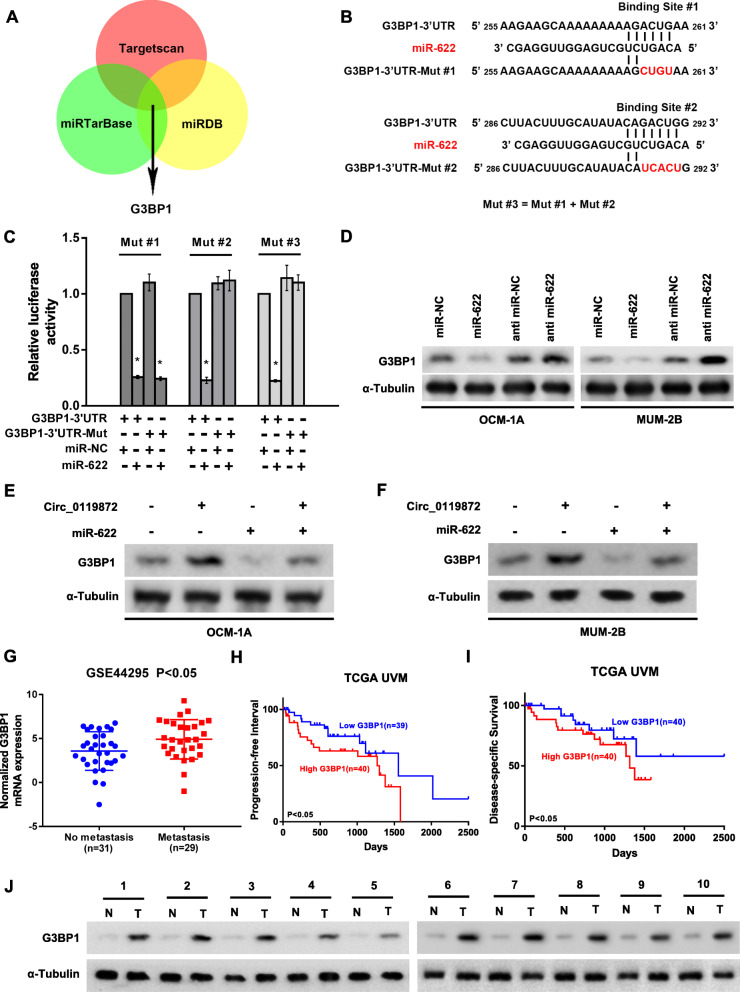
MiR-622 directly targets the G3BP1 3’UTR. **a** The Venn diagram shows that G3BP1 is commonly predicted by TargetScan, miRDB and miRTarBase. **b** Schematic diagram of two potential binding sites of miR-622 and the G3BP1 3’UTR. **c** Dual-luciferase reporter assay shows that miR-622 directly targets the G3BP1 3’UTR by binding to site #2. **d** The protein levels of G3BP1 detected by western blotting in miR-622-overexpressing and knockdown cells. **e** and **f** Circ_0119872 and miR-622 can co-regulate the protein level of G3BP1. **g** G3BP1 expression in the GSE44295 dataset. **h** and **i** Progression-free survival and disease-specific survival in the TCGA UVM dataset with high versus low levels of G3BP1 mRNA. J. G3BP1 protein expression in 10 UM tissues (T) and paired adjacent non-tumorous tissues (N). Bar graphs show the statistical analysis of three independent experiments (* *P* < 0.05)

In an in silico study using three bioinformatics algorithms, TargetScan (http://www.targetscan.org/) [8], miRDB (http://mirdb.org/) [9] and miRTarBase (http://mirtarbase.mbc.nctu.edu.tw/php/index.php) [10], we found that G3BP1 may be a target of miR-622 (Fig. 5a), and there were two potential binding sites in the G3BP1 3’UTR. To define which binding site was functional, we constructed separate plasmids with these two binding sites mutated to be used in the dual-luciferase reporter assay (Fig. 5b). The results confirmed that miR-622 directly targeted the G3BP1 3’UTR and that binding site #2 was the functional site since transfection of miR-622 mimics strongly reduced the activity of the luciferase reporter carrying the wild-type G3BP1 3′-UTR compared to mimic NC, while the luciferase reporter with G3BP1 3′-UTR-Mut#2 was unaffected by overexpression of miR-622 (Fig. 5c). Western blotting assays also demonstrated that upregulating miR-622 could suppress the protein level of G3BP1 (Fig. 5d). Furthermore, by detecting the level of G3BP1 following co-transfection of circ_0119872 and miR-622 mimics into UM cells, we found that they can co-regulate the expression of G3BP1 in UM cells (Fig. 5e and f).

Moreover, expression data downloaded from the GEO database showed that G3BP1 is significantly upregulated in UM patients with metastasis compared to those without metastasis (Fig. 5g), and higher expression of G3BP1 is correlated with worse prognosis according to the expression data acquired from TCGA UVM (Fig. 5h and i). Our experiments also showed that G3BP1 was upregulated in UM tissues compared with normal uvea tissues (Fig. 5j).

### G3BP1 promotes UM cell proliferation and angiogenesis

**Fig. 6:**
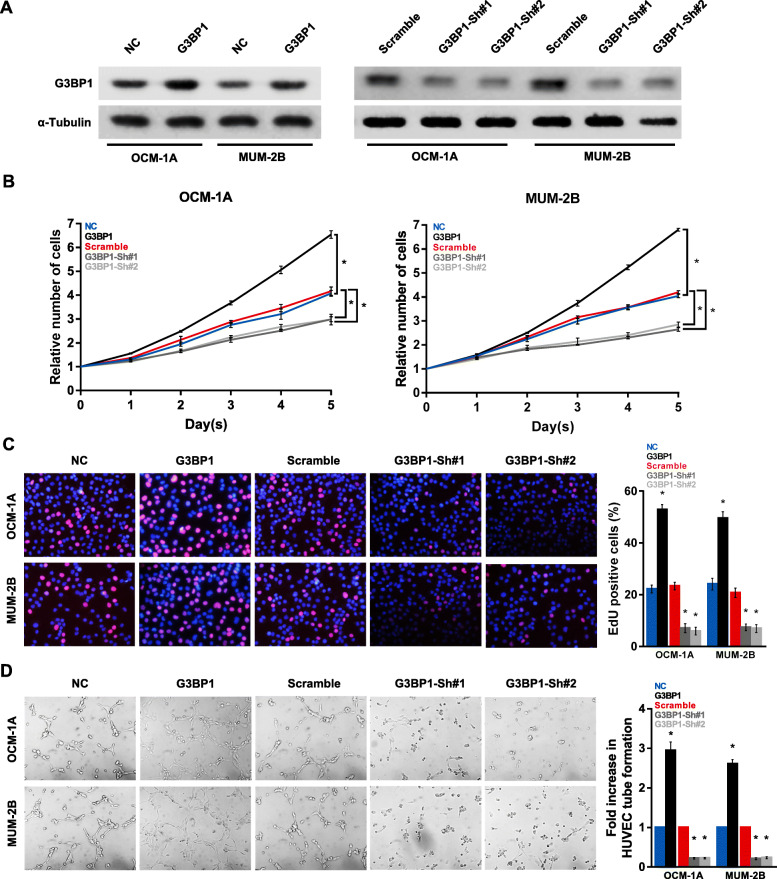
G3BP1 promotes the proliferation and angiogenesis of UM cells. **a** The expression levels of G3BP1 in OCM-1A and MUM-2B cells stably transfected with G3BP1 overexpression or knockdown vector and the corresponding negative controls were detected by western blotting. **b** G3BP1 promotes the proliferation of UM cells, as determined by the CCK-8 assay. **c** Representative micrographs (left panel) and quantification (right panel) of EdU incorporation in UM cells as indicated. DAPI was used as a DNA/nuclear stain. **d** Representative images (left panel) and quantification (right panel) of HUVECs cultured on Matrigel-coated plates with conditioned medium from UM cells

To clarify the effects of G3BP1 on UM cell biofunction, we constructed G3BP1-overexpressing (G3BP1) and G3BP1 knockdown (G3BP1-Sh#1 and G3BP1-Sh#2) cell lines (Fig. 6a). The results of EdU and CCK-8 assays demonstrated that high G3BP1 expression levels promoted cell proliferation, while silencing of G3BP1 expression inhibited cell proliferation in both OCM-1A and MUM-2B cells (Fig. 6b and c). The HUVEC tube formation assay also indicated that cells with ectopic G3BP1 levels exhibited significantly increased angiogenesis (Fig. 6d). Taken together, these results indicate that G3BP1 plays an oncogenic role in promoting UM progression.

### Circ_0119872 activates Wnt/β-catenin and mTOR signalling pathways by regulating G3BP1 in UM

**Fig. 7:**
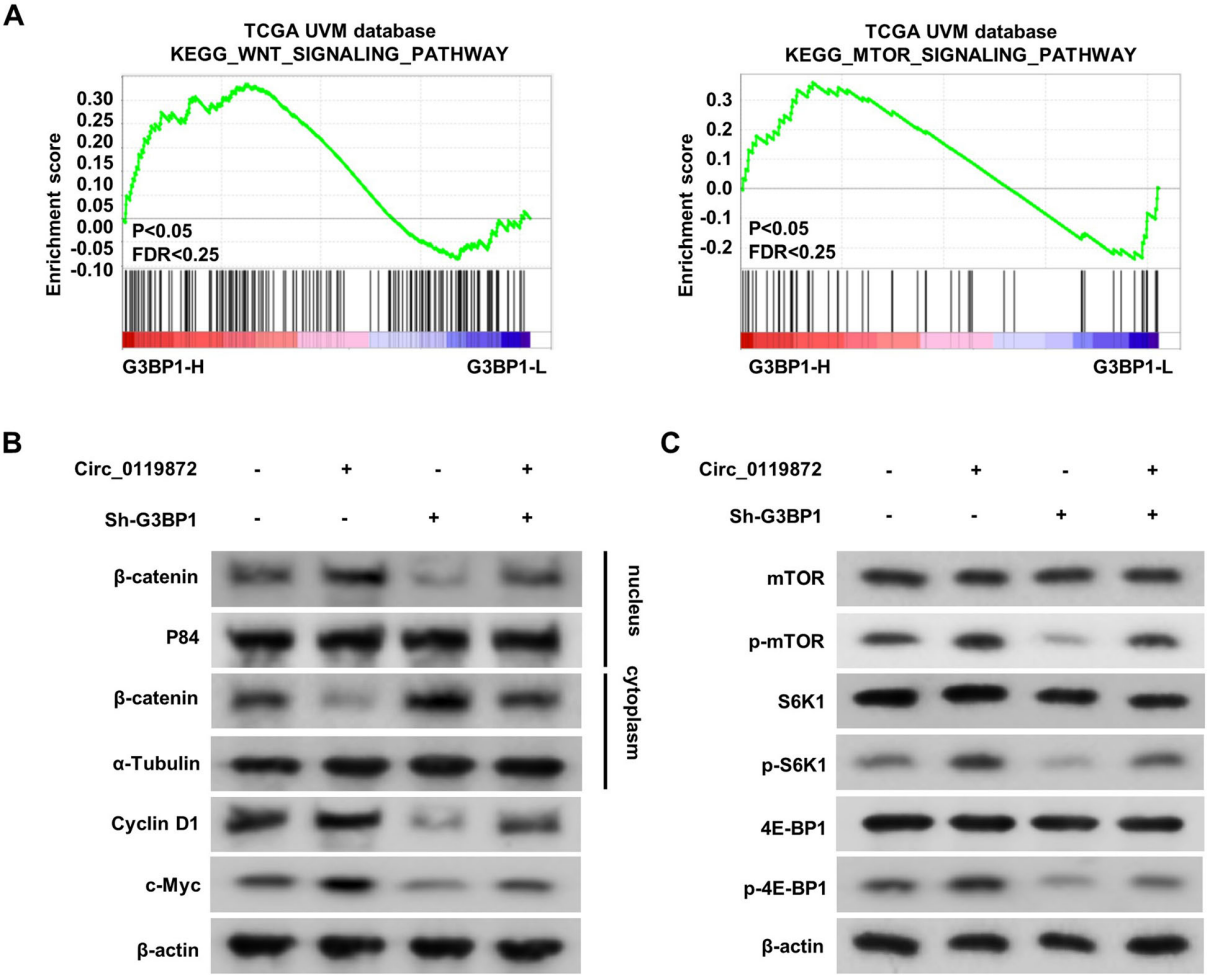
Circ_0119872 activates Wnt/β-catenin and mTOR signalling pathways by regulating the protein level of G3BP1. **a** GSEA plot showing that G3BP1 expression is positively correlated with Wnt/β-catenin and mTOR signalling pathways. **b** Western blotting analysis of the expression of β-catenin in the nucleus, cyclin D1 and c-Myc. **c** Western blotting analysis of the phosphorylation of mTOR, S6K1, and 4E-BP1 in cells as indicated

To further elucidate the molecular mechanism of G3BP1-regulated UM cell biofunction, gene set enrichment analysis (GSEA) was performed. The results indicated that G3BP1 overexpression was positively associated with the activation of Wnt/β-catenin and mTOR signalling pathways (Fig. 7a). Knowing that the expression of G3BP1 could be regulated by circ_0119872, western blotting was performed to clarify the role of circ_0119872 in these two pathways. As shown in Fig. 7b, the ectopic expression of circ_0119872 resulted in increased expression of β-catenin in the nucleus and enhanced the expression of cyclin D1 and c-Myc. In addition, the overexpression of circ_0119872 promoted the phosphorylation of mTOR, S6K1, and 4E-BP1 (Fig. 7c). Moreover, this effect was reversed by knocking down the expression of G3BP1. These results suggest that circ_0119872 activates Wnt/β-catenin and mTOR signalling pathways by regulating G3BP1 expression.

## Discussion

UM is the most common primary intraocular malignancy [11]. Primary disease is efficiently controlled by surgery or radiation therapy, but approximately half of UMs develop distant metastasis mostly to the liver [12]. Therefore, effective early diagnosis and treatment methods are urgently needed. In the past, ncRNAs were considered “evolutionary junk”, but increasing evidence suggests that they have a substantial impact on several molecular mechanisms [13]. The role of dysregulated ncRNAs in the proliferation, migration, invasion, and angiogenesis of cancer cells has generated significant scientific interest [14]. CircRNAs, as abundant stable ncRNAs, have been demonstrated to play a prominent role in UM development. Yang et al. explored, for the first time, the abnormal expression of circRNAs in UM and described the expression profile of circRNAs [6]. However, the specific underlying molecular mechanism of their association with UM development has not been fully explored.

In the current study, we investigated the effect of the novel circRNA circ_0119872 on the development of UM and identified a new regulatory mechanism of circ_0119872/miR-622/G3BP1 signalling. Our results indicate that circ_0119872 acts as a molecular sponge for miR-622 to weaken its inhibitory effect on the downstream target gene G3BP1 and then activates Wnt/β-catenin and mTOR signalling pathways to promote the metastatic potential of UM cells. This study is the first to reveal the function, mechanism and clinical implications of circ_0119872 in human UM.

It is well known that circRNAs may act as miRNA sponges to regulate the expression of target genes [15] and influence the development of various types of cancer [16]. For example, the circRNA circ_0008532 was identified as an oncogene in bladder cancer by sponging miR-155-5p and miR-330-5p [17]. Chen et al. demonstrated that circ_0000527 sponged miR-646 to regulate the expression of BCL-2, promoting the viability, migration and invasion of retinoblastoma cells [18]. However, circRNAs must possess the following characteristics before they can act as miRNA sponges. First, they are derived from one or more exons of known protein-coding genes through back-splicing [19]. Second, the subcellular location of these circRNAs is predominantly in the cytoplasm, which indicates that they occupy the same space as miRNAs. Finally, circRNAs harbouring putative miRNA binding sites could be potential candidates for miRNA sponges [20]. It should be noted that not all circRNAs can act as miRNA sponges. Some circRNAs with short lengths can be packaged into exosomes and function as cancer biomarkers [21]. Intronic and exon–intron RNAs, which are mainly localized in the nucleus, have been reported to regulate the expression of their parental genes via specific RNA–RNA interactions [22]. To better understand the regulatory mechanism of circ_0119872 in UM, we explored its genomic structure and performed RNA-FISH to determine its subcellular location. We found that circ_0119872 is derived from exon 4 and exon 5 of RASGRP3 and is located mainly in the cytoplasm. In addition, bioinformatics prediction, RNA pull-down and luciferase reporter assays showed that both circ_0119872 and the G3BP1 3′ UTR can bind with miR-622 in a reverse complementary manner. In vitro and in vivo experiments further indicated that circ_0119872 and miR-622 can co-regulate cell biofunction in UM and the expression of G3BP1. In summary, this study reveals a circ_0119872/miR-622/G3BP1 axis in UM.

G3BP1, an SH3 domain-binding protein, consists of the following 5 domains (arranged from the N- to C-terminus): a nuclear transport factor 2-like (NTF2-like) domain, an acid-rich domain, proline (PxxP) motifs, an RNA recognition motif (RRM), and a rich in arginine-glycine (RGG) box. In addition, G3BP1 has been reported to be involved in regulating various signalling pathways [23, 24] and plays a vital role in tumour development and progression [25]. Evidence shows that G3BP1 promotes tumour progression and metastasis through the IL-6/G3BP1/STAT3 signalling axis in renal cell carcinoma [26]. Loss of G3BP1 suppresses the proliferation, migration, and invasion of oesophageal cancer cells via inactivation of the Wnt/β-catenin and PI3K/AKT signalling pathways [27]. In addition, a study performed by Xiong et al. demonstrated that silencing G3BP1 inhibits the activation of the transforming growth factor (TGF)-β/Smad signalling pathway in gastric cancer [28]. However, the biological functions of G3BP1 in UM have not been investigated. Our study, for the first time, indicates that G3BP1 is significantly increased in UM tissues and that overexpression of G3BP1 leads to enhanced development in UM. Furthermore, circ_0119872 alleviates the inhibitory effect of miR-622 on G3BP1 and then activates Wnt/β-catenin and mTOR signalling pathways. Together, these findings reveal a crucial link between G3BP1, Wnt/β-catenin and mTOR signalling pathways during UM development.

## Conclusions

In conclusion, we confirmed the upregulation of circ_0119872 in UM tissues and its positive effects on UM cell biofunction. Moreover, circ_0119872 acts as a sponge for miR-622 to reduce the inhibitory effect on G3BP1 and thus enhances the expression of G3BP1 and promotes the activity of the downstream Wnt/β-catenin and mTOR signalling pathways. Overall, our study clarifies that circ_0119872 acts as an oncogene by targeting the miR-622/G3BP1 axis and provides a new target for the diagnosis and treatment of UM.

## Supplementary Information

**Additional file 1: Table S1.** The sequences of primers, oligonucleotides and probes used in this study

**Additional file 2: Table S2.** Top 10 significantly down-regulated and up-regulated circRNAs in UM tissues ranked by FC

**Additional file 3: Figure S1.** The effect of circ_0119872 on UM cells migration/invasion and metastasis

## Data Availability

The datasets used and/or analysed during the current study are available from the corresponding author on reasonable request.

## Abbreviations

UM: Uveal melanoma
GSEA: Gene Set Enrichment Analysis
TCGA: The Cancer Genome Atlas
GEO: Gene Expression Omnibus
G3BP1: GAP SH3 Domain-Binding Protein 1
PCR: Polymerase Chain Reaction
FISH: Fluorescence in situ hybridization
RASGRP3: RAS guanyl releasing protein 3
