# Supplementary figures and images for: Circ_0119872 promotes uveal melanoma development by regulating the miR-622/G3BP1 axis and downstream signalling pathways
Source: J Exp Clin Cancer Res. 2021 Feb 12;40:66. doi: 10.1186/s13046-021-01833-w (PMC7881613; doi:10.1186/s13046-021-01833-w)

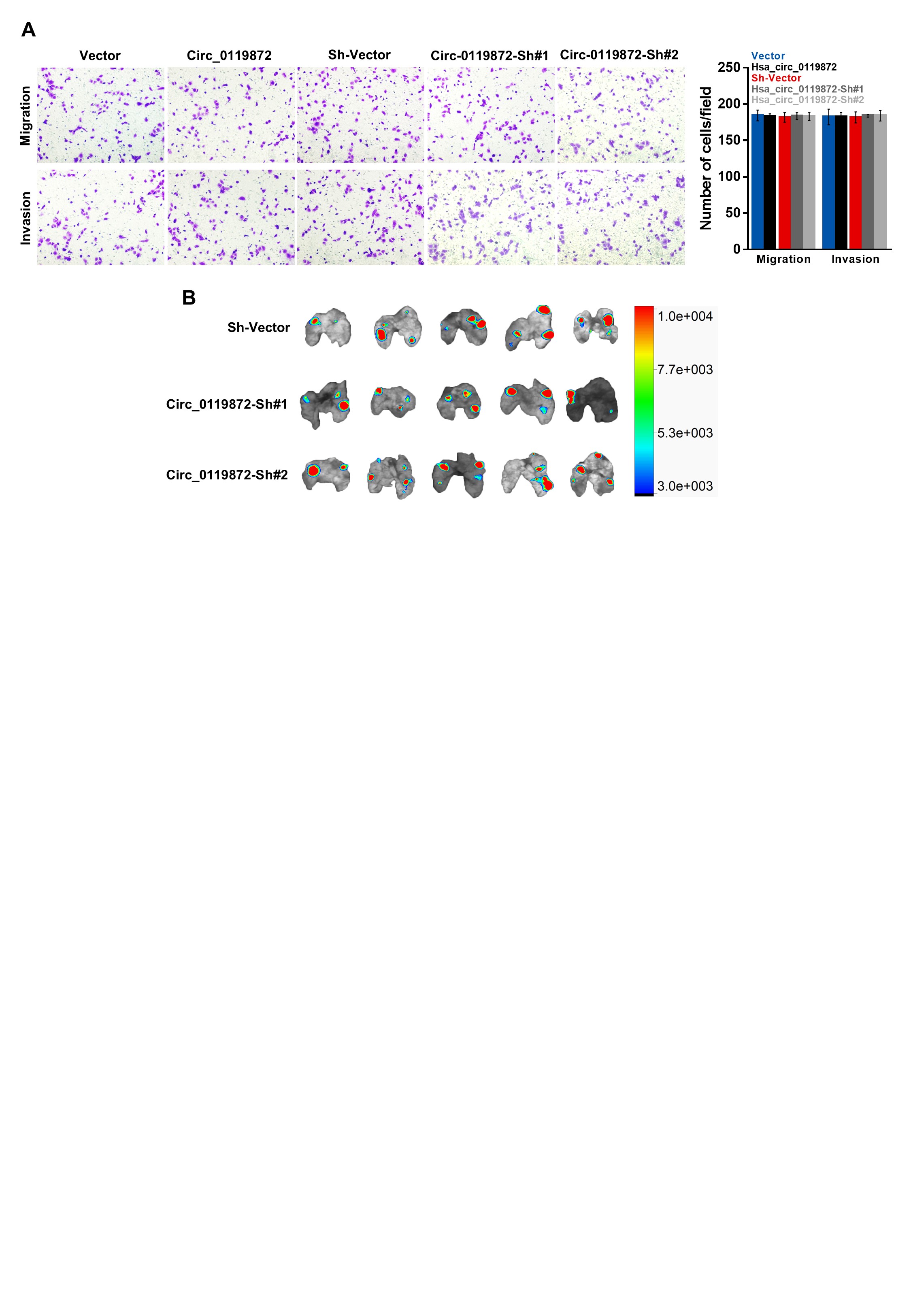

Supplement: Supplementary file 3 — Additional file 3: Figure S1. The effect of circ_0119872 on UM cells migration/invasion and metastasis [file 13046_2021_1833_MOESM3_ESM.jpg]
